# Lipid-Based Nanoparticles as a Potential Delivery Approach in the Treatment of Rheumatoid Arthritis

**DOI:** 10.3390/nano8010042

**Published:** 2018-01-15

**Authors:** Shih-Yi Chuang, Chih-Hung Lin, Tse-Hung Huang, Jia-You Fang

**Affiliations:** 1 Research Center for Food and Cosmetic Safety and Research Center for Chinese Herbal Medicine, Chang Gung University of Science and Technology, Kweishan, Taoyuan 333, Taiwan; clemencechuang@gmail.com; 2 Center for General Education, Chang Gung University of Science and Technology, Kweishan, Taoyuan 333, Taiwan; chlin@mail.cgust.edu.tw; 3 Department of Traditional Chinese Medicine, Chang Gung Memorial Hospital, Keelung 204, Taiwan; huangtsehung@gmail.com; 4 School of Traditional Chinese Medicine, Chang Gung University, Taoyuan 333, Taiwan; 5 School of Nursing, National Taipei University of Nursing and Health Sciences, Taipei 112, Taiwan; 6 Pharmaceutics Laboratory, Graduate Institute of Natural Products, Chang Gung University, Kweishan, Taoyuan 333, Taiwan; 7 Chinese Herbal Medicine Research Team, Healthy Aging Research Center, Chang Gung University, Kweishan, Taoyuan 333, Taiwan; 8 Department of Anesthesiology, Chang Gung Memorial Hospital, Linkou, Taoyuan 333, Taiwan

**Keywords:** rheumatoid arthritis, lipid nanoparticle, liposome, drug delivery

## Abstract

Rheumatoid arthritis (RA), a chronic and joint-related autoimmune disease, results in immune dysfunction and destruction of joints and cartilages. Small molecules and biological therapies have been applied in a wide variety of inflammatory disorders, but their utility as a therapeutic agent is limited by poor absorption, rapid metabolism, and serious side effects. To improve these limitations, nanoparticles, which are capable of encapsulating and protecting drugs from degradation before they reach the target site in vivo, may serve as drug delivery systems. The present research proposes a platform for different lipid nanoparticle approaches for RA therapy, taking advantage of the newly emerging field of lipid nanoparticles to develop a targeted theranostic system for application in the treatment of RA. This review aims to present the recent major application of lipid nanoparticles that provide a biocompatible and biodegradable delivery system to effectively improve RA targeting over free drugs via the presentation of tissue-specific targeting of ligand-controlled drug release by modulating nanoparticle composition.

## 1. Introduction

**Figure 1:**
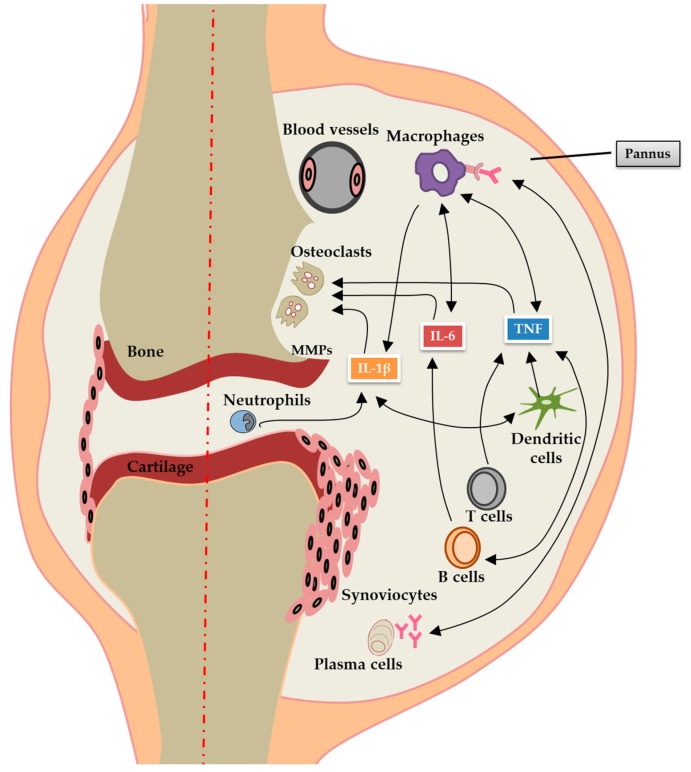
Pathogenesis of RA: pannus formation and systemic inflammation. Inflammation in RA is caused by activation of B cells, T cells, plasma cells, neutrophils, dendritic cells and macrophages, which releases proinflammatory cytokines such as TNF-α, IL-1β, and IL-6. These cytokines cause local joint damage through increased production of MMPs and activation of osteoclasts. TNF-α, IL-1β, and IL-6 also leak out to the blood stream resulting in systemic inflammation. TNF, tumor necrosis factor; IL, interleukin; RA, rheumatoid arthritis; MMPs, matrix metalloproteinases.

Rheumatoid arthritis (RA) is an autoimmune-mediated joint-based chronic inflammatory disease with a prevalence in the population ranging from 0.5% to 1%. It can present at any age and is more prevalent in women than in men. The clinical pathology of RA causes progressive articular destruction, and its associated comorbidities of vascular, metabolic, bone, and psychological problems have been extensively reviewed [1,2]. RA pathogenesis is a multistep process, initially starting outside the joints. It involves the activation of innate immune cells (dendritic cells, macrophages, and neutrophils) by one or more strong environmental effects, with activation of the inflammatory cascade, as well as aberrant T cell and B cell cross-regulation. This culminates in the production of autoantibodies to cells of the adaptive immune system that recognize a range of post-translationally modified proteins with citrulline residues [3]. Moreover, the development of RA is considered to appear when genetic and environmental factors interact and trigger immunological changes leading to an inflammatory arthritis [4,5]. In the last decades, we have known that there is a genetic susceptibility factor in RA which contributes 60% to the development of this disease. Most of the immune effectors or regulatory gene products in RA have been well characterized through a genome-wide association study [6], providing novel insight into RA pathogenesis. That the prominent genes of RA risk loci encode major histocompatibility complex (MHC) class II molecules (e.g., the *HLA-DRB1* allele) for T-cell recognition of the autoreactive peptide has been confirmed in patients who are positive for rheumatoid factor or anti-citrullinated protein antibody (ACPA). Many other identified risk alleles in ACPA-positive RA are functionally involved in immune regulation, implicating the intracellular inflammatory pathways (e.g., *TRAF1-C5*, *c-REL PTNP22*, and *STAT4*). Additionally, post-translational citrullination enzymes (e.g., *PADI-4*, which acts in the post-translational modification of peptidylarginine to citrulline) might alter the threshold for immune activation or failed regulation [7,8,9,10,11,12]. Many environmental factors, including exposure to tobacco smoke, Epstein-Barr virus (EBV) infections, hormones, obesity, alcohol, as well as gene—environment interactions have been associated with increased risk for RA [13]. Smoking is the most widely studied and recognized environmental risk factor for development of RA [14]. It has been observed that the use of tobacco modulates cell death, resulting in stimulating the population of antigen presenting cells present in the lungs and facilitating the development of autoimmunity. Furthermore, tobacco smoking contains very high concentrations of free radicals to interact with DNA that may cause mutation or gene activation. The traditional paradigm for RA involved synovitis and was characterized by the infiltration of inflammatory cells, synovial hyperplasia, autoantibody production and excess synovial fluid, resulting in joint swelling, pain, and progressive stiffness and also leading to the destruction of articular cartilage and bone erosion. During this process, the synovial macrophages, B cells, fibrocytes, synoviocytes, CD4^+^, and CD8^+^ T cells produce several proinflammatory cytokines, including tumor necrosis factor-alpha (TNF-α), interleukin-1 beta (IL-1β), and IL-6. It is evident that proinflammatory cytokines and intracellular kinases play an important role in the pathogenesis of this disease, and the activated synoviocytes exhibit invasive growth into the joint cartilage. Furthermore, both TNF-α and IL-6 induce synovial cells to release tissue-degrading matrix enzymes, particularly members of metalloproteinase, and TNF-α stimulates abnormally the differentiation and proliferation of osteoclasts, which are responsible for bone erosions [1,15] (Figure 1).

Curing RA is still out of our reach, and the induction of immunologic tolerance will not be achieved until the autoantigens or cytokine cascades in RA have been fully identified. Earlier, a broad spectrum of anti-rheumatic drugs was available to reduce painful symptoms in patients and to slow down the progression of the disease. Currently, therapeutic strategies for RA include nonsteroidal anti-inflammatory drugs (NSAIDs) [16]; corticosteroids [17]; disease-modifying anti-rheumatic drugs (DMARDs) [18], biological drugs [19], and natural agents [20]. These are summarized in Table 1. Biological drugs, also called biological DMARDs, are targeted treatments that block the actions of specific cytokines or immune regulators. Currently, several classes of biological DMARDs are available for clinical application, including (1) the TNF-α inhibitors; (2) the T cell activation inhibitor via anti-CD80/86 inhibition; (3) the anti-CD20 agent that causes B cell depletion via anti-CD20 inhibition; (4) the IL-6 receptor blocker; and (5) the intracellular kinase inhibitor. Previously, RA patients were initially treated with NSAIDs to reduce pain and joint swelling. Currently, patients are treated with DMARDs more aggressively to prevent joint damage. Depending on the symptoms and stage of the disease, using a combination of several DMARDs or a DMARD plus biological drugs, such as tofacitinib plus methotrexate, is thought to have fewer side effects than using other DMARDs. This approach has been demonstrated to have a favorable therapeutic outcome [21,22]. The natural agents, including curcumin, resveratrol, guggulsterone and withanolide, are some of the polyphenols that have been tested for the treatment of arthritis [23]. Although conventional therapy may achieve a therapeutic effect to a certain extent, it still comes with high risks of therapeutic intolerance and toxic effects induced by dose escalation. Patients indeed need advanced therapies with minimized side effects. Nanoparticles represent a novel drug delivery system that can be engineered to harness optimal targeting of drugs to a specific site for cells and tissues and to have more drug-loading capacity, allowing improved pharmacokinetics, safe and effective drug delivery, as well as enhanced bioavailability of therapeutics [24,25]. Compared to the traditional drugs, drug-loaded nanoparticle carriers present several advantages, including improved delivery of insoluble drugs, selective recognition of the target cells and lower systemic side effects, protection of drug degradation, controlled release of the drugs, promotion of the drug transport across the biomembrane, and combined diagnostic tools as theranostic agents.

Generally, nanoparticles are found in a size range between 10 to 1000 nm, depending on different matrix materials, and have varying surface characteristics as well as mechanical and physicochemical properties. The application of nanoparticles in drug delivery in the treatment of various diseases has already been studied. Many studies have focused on the use of nanoparticles in the field of autoimmunity [24,26]. This is because nanoparticles can be designed to be highly selective for cells and allow a slow release of anti-inflammatory agents, causing reduction of systemic toxicity and improvement of the distribution of these agents in the body [27]. Dose escalation due to the nonselective activity of the drugs often limits the application in current RA therapy. In RA therapy, nanotechnology-based approaches have been demonstrated to be particularly useful to resolve this problem. This is because nanoparticulate systems are capable of reducing the toxic side effects of chemotherapeutic agents while enhancing their anti-inflammatory efficacy. Generally, anti-inflammatory therapeutics are quite toxic to both inflamed cells and normal cells; this represents one of the major problems as their use can be limited by their toxicity. However, by implementing different strategies, such as passive and active targeting, the incorporation of anti-inflammatory drugs into nanoparticles can improve their specificity to inflamed cells and tissues [28,29]. In the following sections, examples of the recent nanotechnology-based approaches to RA treatment will be reviewed and then presented in Table 2.

Passive targeting of the nanocarriers is based on the properties of the delivery system. Its effectiveness at specifically accumulating the drugs at a targeted site and avoiding nonspecific distribution depends on the status of the disease. The passive targeting strategy for cancer is based on the enhanced permeability and retention (EPR) effect in abnormal leaky vessels, which ensures extravasation and retention of nanoparticles into the interstitial space of the inflamed tissue [29]. In this regard, similar to cancer, abnormal vessels and inflammatory cell infiltration at the affected sites are also the remarkable characteristics of RA. Thus, the leaky vessels of RA are usually utilized as the treatment targets for selective drug delivery [30]. Formation of endothelial gaps in RA allows for the leakage of plasma into the injured sites, followed by the recruitment of monocytes and the overexpression of inflammatory mediators. Through the EPR effect, the appropriately sized nanoparticles would permeate through the gaps among the endothelial cells into the synovial tissue, and they would be trapped there for slow drug release. Several reports suggest that inflamed tissues in RA models also present enhanced vascular permeability, which allows small, long-circulating drug carrier systems to extravasate at these sites via the EPR effect. Subsequently, they are retained in the extravascular space, with a large portion being taken up by macrophages in the synovial layer [31,32]. This indicates that the size of the nanoparticles is a decisive factor in the passive targeting process. During this process, wide gaps up to 700 nm were formed among the inter-endothelial cell junctions [33]. Previous studies had suggested that nanoparticles in a size range between 20 and 250 nm can accumulate inside the inflammatory space, because leaky blood vessels in the pathogenesis of RA are made up of a porous endothelial lining with larger pore sizes between the endothelial barriers than found in normal blood vessels [34,35,36,37,38,39,40,41,42,43]. Furthermore, by coupling of targeting structures to the liposomal membrane, specific cell populations can be targeted effectively in the pathological site. Polyethylene glycol (PEG) or poly(ethylene oxide) is helpful for improving the effectiveness of passive targeting and also allows for an increase in nanoparticle circulation time by reducing opsonin adhesion, thus lowering nanoparticle recognition by the reticuloendothelial system (RES) [44]. Combination of passive targeting and the EPR effect make the use of long-circulating liposomes attractive for improving the therapeutic index of antirheumatic drugs.

Apart from passive targeting, nanoparticulate drug-delivery systems can be modified to be more selective toward the specific surface of the targeted cells by means of active targeting. In other words, the active targeting strategy is able to further improve the therapeutic efficacy through the high affinity of ligands to the receptors expressed under specific circumstances or localized on the surface of particular cells such as the activated macrophages, T cells, and vascular endothelial cells found in the nidus of RA. In active targeting, specific ligands recognized by cells at the diseased site are conjugated to the surface of the nanoparticles and further reduce drug retention in normal tissues. For example, the receptor, such as the folate receptor (FR) and α_V_β_3_ integrins should be overexpressed on inflammation-associated cells and not expressed on normal cells [45,46,47]. The internalization of a ligand-receptor complex usually occurs via receptor-mediated endocytosis and envelops the ligand-receptor complex, forming an endocytic vesicle [48,49,50,51]. For active-targeting drug delivery, the final destination of the receptors and ligands may not only be the main determinant of the efficacy but also depend on the chemical properties of the drugs, such as net ionic charge, log *p* value, and amphiphilicity. Targeting of macrophages through the accumulation of nanoparticles at sites of inflammation by nanoparticulate systems has been proved as a powerful approach for the treatment of RA [52,53].

## 2. Anti-RA Activity of Lipid-Based Nanoparticles

**Figure 2:**
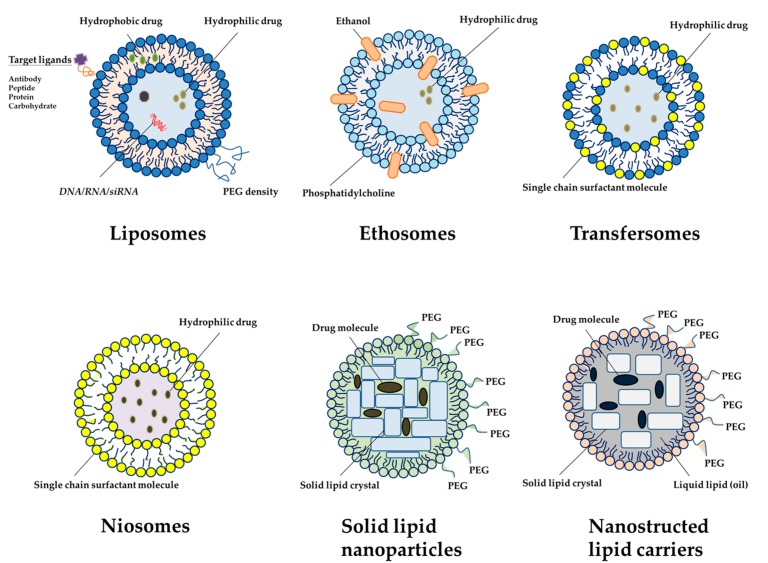
An overview of different types of lipid nanocarrier systems used for the treatment of arthritic diseases (plotted in Table 3). In brief, liposomes are typically composed of natural phospholipids, a major component of most biological membranes. Ethosomes are lipid vesicles composed of phospholipids and a large amount of ethanol. Transfersomes are liposomes with deformable properties enabling the stratum corneum to pass and penetrate deeply into the skin. Solid lipid nanoparticles are constituted by a mixture of solid lipids dispersed in inner cores. Nanostructured lipid carriers are formed by the mixture of solid lipids and liquid lipids in the cores. PEG: Polyethylene glycol.

Lipid-based nanocarriers are composed of physiological lipids; hence, they are well tolerated, usually nontoxic, and are degraded to a nontoxic residue. Over the past ten years, research has been focused on new approaches using novel lipid-based nanocarriers including liposomes, niosomes, ethosomes, transfersomes, solid lipid nanoparticles (SLN), nanostructured lipid carriers (NLC) and lipid nanoemulsions for safe and effective delivery of anti-inflammatory drugs [28] (Figure 2). Table 3 summarizes the application of lipid-based nanoparticles in the treatment of RA.

Liposomes are the first examples of the developed lipid-based carriers that are characterized to be non-toxic, flexible, biocompatible and completely biodegradable [55]. They are mainly composed of phospholipid bilayer vesicles containing phosphatidylcholine and phosphatidylethanolamine, the most common phospholipids found in nature, with other membrane bilayer constituents, such as cholesterol and hydrophilic polymers around each liposomal vesicle [56]. Cholesterol, an important component in the preparation of liposomes, helps to decrease the fluidity of the liposomal membrane bilayer, reduce the permeability of water-soluble molecules through the liposomal membrane, and improve the stability of the liposomal membrane in biological fluid, such as blood and synovial fluid [57]. Apart from cholesterol, a small fraction of polymers containing hydrophilic groups, especially PEG, is conjugated to the surface of liposomes and is often used for its stealth function in nanoparticle formulations that minimizes undesired phagocytic clearance; it can also interfere with the ability of nanoparticles to interact with and be internalized by target cells because it is a hydrophilic and flexible polymer [58]. Although PEGylation improves the circulation time and the efficient transport of liposomes to the tissue, the incorporation of PEG to the surface of the liposomes may reduce the binding and the uptake of liposomes by cells due to the steric hindrance provided by PEG [59,60]. However, as described in previous sections, PEGylation can be optimized to provide increased delivery to target cells by overcoming extracellular barriers through the incorporation of target ligands to the terminal ends of the PEG chains [61]. The presence of hydrophilic PEG moieties on the nanocarrier surface may reduce the escape of the drug from the endosomal compartment and its entry into the cytoplasm. This challenge can be resolved by coating PEG with pH sensitivity, enzyme-cleavable linkage, and interference with PEGylation, leading to the liberation of PEG moiety from the nanoparticulate surface in the intracellular vesicles [62]. It has been reported that loperamide HCl liposomal gel was topically applied twice daily from day 0 at the same time as immunization. The histological results exerted the analgesic and anti-inflammatory effects exclusively in peripheral painful inflamed tissue over 48 h in rats with a complete Freund’s adjuvant (CFA) arthritis model [54]. Trif et al. [63] developed lactoferrin, a glycoprotein that possesses anti-inflammatory and antimicrobial activities, which they used to encapsulate liposomes into the collagen-induced arthritis mouse model to reduce the inflammation. The results showed that the positively charged liposomes, after injection for 2 h, were more efficient in prolonging the residence time of lactoferrin in the inflamed joint as compared with other liposomes, suggesting that the entrapment of lactoferrin in positively charged liposomes could modify its pharmacodynamic profile and be of therapeutic benefit in the treatment of RA and other local inflammatory conditions.

Niosomes play an important role owing to their nonionic properties, which are formed by the hydration of nonionic surfactant with cholesterol, and can be used as an important tool for immunological selectivity, low toxicity and good stability to protect the incorporated active moiety [64]. Their properties may vary according to changes in their size, lamellarity, and surface charge. Niosomal inclusion can enhance the circulation time of the therapeutic molecules along with high stability. Niosomal drug delivery is widely reported in the literature for various serious maladies including RA and cancers [64]. Transdermal delivery of ursolic acid-loaded niosomes showed enhanced skin permeability compared with the control formulation with an enhancement ratio of 4.84 [65]. In vivo efficacy in rats showed that ursolic acid-loaded niosomes had a significant improvement vis-a-vis oral ursolic acid formulation and the conventional gel system. Luteolin-loaded niosomes were prepared using different nonionic surfactants and were characterized for in vitro and in vivo anti-arthritic activity, providing both the improved entrapment efficiency and enhanced transdermal flux across the rat skin [66]. The in vivo bioactivity studies revealed that the prepared luteolin-loaded niosomes were able to provide good anti-arthritic activity compared to the standard gel. The rat paw volume showed a depriving effect with an enhanced red blood cell (RBC) count and a decreased white blood cell (WBC) count. The aforementioned observations make it evident that luteolin-loaded niosomes were effective in arthritis management.

Ethosomes are a modification of classical liposomes and are composed of phospholipids, a high concentration of ethanol up to 45% *w*/*w*, and water. It was reported to be superior over classical liposomes for transdermal drug delivery because they were smaller and had negative zeta potential and higher entrapment efficiency [67]. The ethosomal formulation of topically administered capsaicin was evaluated for bio-efficacy in arthritic rats. The results revealed a significant reduction of rat paw edema along with promising antinociceptive action [68]. No predictable signs of toxicity such as skin irritation were observed. Tetrandrine-loaded ethosomes by topical application were used to explore the feasibility of ethosomes for improving the anti-arthritic efficacy in the adjuvant-induced arthritis model [69]. Ex vivo permeation and deposition behavior demonstrated that the drug flux across the rat skin and the deposition of the drug in the rat skin for ethosomes was 2.1- and 1.7-fold higher than that of liposomes, respectively. Transfersomes are elastic liposomes composed of phosphatidylcholine and an edge activator. The skin permeation and penetration of these elastic vesicles result from a synergic mechanism between the carrier properties and the permeation enhancement ability. Transfersomes can cross the skin layers by different mechanisms depending on their composition, in which these vesicles maintain their intact structure or fuse and mix with skin lipids [70]. They can encapsulate a wide range of drug molecules and easily change their shape to cross pathways 5 to 10 times narrower than their own diameter, thus providing greater drug penetration. These unique properties make them superior in drug-delivery efficiency [71]. Celecoxib, loaded into transfersomes containing soy phosphatidylcholine mixed with sodium deoxycholate, has been shown to be a therapeutically effective drug-delivery system for the treatment of rheumatoid arthritis due to its higher permeation over conventional gel in rat skin’s arthritic mode [72]. The transfersomal vesicular system was employed for the topical administration of capsaicin in experimental rats [73]. Capsaicin-loaded transfersomes were demonstrated to exhibit better skin penetration into the rat skin. The in vivo anti-arthritic activity study showed superior inhibitory activity compared to the marketed Thermagel^®^ at the same dosage level. This could probably be due to the lesser permeability of Thermagel^®^ across the dermal barriers. Garg et al. [74] developed piroxicam-loaded transethosome using the central composite design. They found that the nanocarriers enhanced drug retention in the skin, drug permeation, and entrapment efficiency in porcine skin as compared to other gel formulations.

In the early 1990s, SLNs, a new class of lipid particle drug carrier, were developed. SLNs are also known as solid lipid nanospheres at room temperature [75,76]. The solid lipid is used as a matrix material for drug encapsulation and can be selected from a variety of lipids, including monoglycerides to triglycerides; glyceride mixtures; and lipid acids. SLNs offer the advantages of physical stability, protection against labile drug degeneration, controlled release, and easy preparation [77]. Moreover, toxicity and acidity issues seem to be not observed in SLNs because the source of lipids used to prepare SLNs is more biocompatible and biodegradable than polymeric materials [78]. A pharmacodynamic study was evaluated for piperine-encapsulated SLN by oral and topical administrations to antigen-induced arthritic rats. The SLNs evoked a significant response, showing a significant reduction in TNF-α in the treated rat, which might be the reason behind the DMARD action of piperine [79]. The ex vivo study using Franz diffusion cell indicated that piperine SLN accumulated in the skin. A study systematically examined the intravenous injection formulation of SLN loaded with actarit, a poor water-soluble anti-rheumatic drug, and found that the area under the curve of the plasma concentration-time (AUC) of actarit-loaded SLNs was 1.88 times greater than that of the actarit in a 50% propylene glycol solution [80]. These results demonstrated that the injectable actarit-loaded SLN was a promising passive targeting therapeutic agent for RA. Albuquerque et al. [81] applied curcumin-loaded SLNs for the treatment of RA. The administration of curcumin-loaded SLN in arthritic rats exhibited a significant decrease in the blood leukocyte count, oxidative stress, TNF-α, C-reactive protein, cyclic citrullinated peptide antibody levels, and radiological alterations in the tibiotarsal joint. In addition, the anti-inflammatory study of complete adjuvant-induced arthritis in rats treated with tripterygium-loaded SLN showed that the nanocarrier group could significantly reduce the rat paw volume and the serum alanine aminotransferase, aspartate aminotransferase, alkaline phosphatase, γ-glutamyl transpeptidase, and albumin levels in serum [82]. The histopathological observation revealed that free tripterygium caused more serious damage to the liver than tripterygium-loaded SLN. These results suggested that the SLN delivery system can enhance the anti-inflammatory activity of tripterygium. Arora et al. [83] applied curcumin-loaded SLN for the treatment of RA. Administration of curcumin-loaded SLN in arthritic rats exhibited a significant decrease in blood leukocyte count, oxidative stress, TNF-α, C-reactive protein, cyclic citrullinated peptide antibody levels, and radiological alterations in the tibiotarsal joint.

NLC, the second generation of lipid nanoparticles—SLN being the first generation—was developed in 1999 by the Müller group to improve the burst-release problem observed in the case of SLN [84]. NLC nanosystems are lipid nanoparticles composed of a solid lipid matrix incorporated with liquid lipid or oil. The solid lipid matrix immobilizes the drug and prevents the particles from coalescing with one another, whereas the liquid oil droplet within the solid matrix can increase the drug-loading capacity of the nanoparticles. Thus, the mixture of lipids allows more drugs to be encapsulated evenly and prevents rapid drug diffusion from the surface of the nanoparticles [85,86]. Recently, a study demonstrated that methotrexate-loaded NLC could be delivered through the transdermal route to the inflamed joints of antigen-induced RA rats [87]. The greater therapeutic efficiency of methotrexate-loaded NLC over conventional gel was shown in the reduced levels of different inflammatory markers and bone-degrading enzymes. Flurbiprofen is used in the treatment of arthritis. However, its multiple dosing due to its short elimination half-life is a concern for such treatment. Flurbiprofen was encapsulated into NLC to evaluate the potential for transdermal delivery and penetration into the skin’s follicles [88]. The bioavailability of flurbiprofen from NLC was more than 1.7-fold that of the commercial gel. Another study showed that methotrexate nanoemulsion was taken up mainly by the liver and the uptake by arthritic joints was 2-fold greater than that by the control joints [89]. The methotrexate nanoemulsion treatment reduced the leukocyte influx into the synovial fluid by nearly 65%; in particular, mononuclear and polymorphonuclear cells were reduced by 47% and 72%, respectively. In another study using nanoemulsion as a diagnostic tool to explore the risk of other diseases in RA patients, LDL-like nanoemulsions labeled with ^14^C-cholesteryl ester and ^3^H-unesterified cholesterol were intravenously injected [90]. The results suggested that RA patients were more efficient in removing low-density lipoprotein (LDL) and protected the LDL plasma fraction against lipoprotein oxidation without the increase of incidence of coronary artery disease.

## 3. Patents for RA Treatment by Nanoparticles

Nanotechnologists have been increasing the implementation of lipid-based nanoparticles as RA therapeutics day by day. Moreover, several related patents have garnered wide attention. Table 4 illustrates the recent advancement in the field for this purpose.

## 4. Conclusions

RA is an autoimmune disease with complex pathogenesis, and it causes bone erosion, deformation, and even physical disability. Although the conventional drug formulation should give an optimum response in RA, neither single drug treatment nor combination therapy has acquired satisfactory outcome, which is accompanied by severe systemic side effects, frequent administration, tolerance from long-lasting administration and high costs. Also with the newer biological therapeutics, there is a need to improve their side effect. To address these issues, extensive attention has been given to the concept of nanoparticles as drug carriers to improve the therapeutic index of drugs. Most of the current studies demonstrate improved efficacy when a drug is administered in a nanoparticle formulation as compared to the free drug, mainly in three aspects: selective accumulation, controlled drug release, and reduced systemic toxicity. The newly developed nanocarriers significantly enhance the therapeutic effectiveness of current drugs for improved RA in experimental models by overall dose reduction and higher local drug localization by passive and active drug targeting. Lipid-based nanoparticles are more advantageous compared to other nanoparticles because of the more biocompatible and biodegradable nature of their constituents relative to the synthetic polymers found in other types of nanoparticles. To date, many anti-arthritic drugs, such as sarilumab (anti-IL-6) [91] and ixekizumab (anti-IL-17) [92], have been developed to block signaling pathways in RA for the application of pharmaceutical and biological cotherapy; they also inhibit the Janus kinase pathway and have already been used in clinical practice. As a result, it is expected that, over time, we will see more new therapeutic targets in biological and small molecule DMARDs. The application of the combination of nanotechnology and biological drugs reflects the popularity of a new therapeutic pattern. In fact, it is believed that the approach of targeting multiple targeted cells and cytokines is a new development direction for future research; it will be achieved through blocking several pathways simultaneously in the pathogenesis of RA.

Although some drug-loaded nanocarriers have been developed for testing cell-based and animal studies, the clinical trials for RA management are still limited. This may be due to the high cost of clinical trials and the unknown side effects that should be identified and explored first. Nanomaterials are thought to elicit more serious adverse effects on organisms compared to materials with larger-sized particles, as their very small size produces a correspondingly higher surface area. Consequently, scientists should pay attention not only to the therapeutic benefits of nanoparticles but also to their adverse effects on both human health and the environment. Caution should be taken in optimizing the feasible conditions of nanomedicine for balancing the effectiveness of anti-RA therapy and tissue damage or toxicity. For employment in human applications, the materials utilized for preparing anti-RA nanotherapeutics should be nontoxic, biodegradable and biocompatible. The materials approved by the US Food and Drug Administration for generally recognized as safe (GRAS) may be the essential choices for the development of these nanocarriers. Presently, a variety of lipid-based nanoparticles encapsulated with drugs are clinically approved and commercially available, while many more formulations are being investigated in different stages of clinical trials or are awaiting approval. However, further studies are still required to optimize their capacity as drug-delivery systems. Lipid-based nanoparticles display the capability to improve the efficacy and safety profile of anti-arthritic drugs and, more importantly, the outcome for RA patients. 

## Figures and Tables

**Table 1:** Current pharmacotherapies in the treatment of Rheumatoid Arthritis.

Therapeutic Classification	Therapeutic Category	Drugs/Agents	Mechanism of Action	Side Effect	Reference
NSAIDs	-	Aspirin, celecoxib, indometacin, ibuprofen	COXs inhibitors, Immunomodulation	Gastrointestinal reaction, dysfunction of kidney, etc.	[16]
Glucocorticoids	-	Dexamethasone, hydrocortisone, prednisone and methylprednisolone	Immunosuppression	Hyperadrenocorticism, infection, hypertension and atherosclerosis, osteoporosis and osteonecrosis, etc.	[17]
DMARDs	-	Methotrexate, hydroxychloroquine, sulfasalazine, clodronate and leflunomide	Immunosuppression, Disease-modifying activity	Myelosuppression, gastrointestinal reaction, dysfunction of liver and kidney, etc.	[18]
Biological agents	Anti-cytokines	Anakinra, Sarilumab, tocilizumab	IL-1 receptor	Infection	[19]
		Sarilumab, tocilizumab	Interlukin-6R inhibitor	Infection, gastrointestinal perforation	
		Sirukumab, olokizumab, siltuximab	Interlukin-6 inhibitor	Infection, gastrointestinal perforation	
		Etanercept, adalimumab, ifliximab, certolizumab pegol, golimumab	TNF-α inhibitor	Infection, tuberculosis	
	Anti-T cell	Abatacept	Co-stimulation inhibitors	Infection, malignancy	
	Anti-B cell	Rituximab	B-cell depletion (anti-CD20)	Infection, hypertension	
	Kinase inhibitors	Baricitinib, tofacitinib	Janus kinase(JAK)1 and 2 inhibitor	Infection	
Natural products	-	Curcumin, Resveratrol, Guggulsterone, Withanolide	IL-6, COX-2, TNF-α	-	[20]

COX: cyloxygenase; JAK: Janus kinase; MMPs: matrix metalloproteinases; TNF: tumor necrosis factor.

**Table 2:** Current nanocarrier system in the treatment of rheumatoid arthritis.

Therapeutic Classification	Drugs/Agents	Nanocarrier System	Mean Size (nm)	Delivery/Target	Model	Reference
NSAIDs	Indomethacin	Polymeric micelles	240	EPR	AIA	[37]
	Aceclofenac	Lysine-liposomes	-	EPR	AIA	[38]
	Indomethacin	Folate-PEG-PAMAM dendrimer	<100	Folate receptor (macrophages)	Patients	[45]
	Indomethacin	Lipid microspheres	150	EPR	AIA	[54]
Glucocorticoids	Dexamethasone	Liposomes	96	EPR	AIA	[39]
	Methylprednisolone	Cyclodextrin polymer	27	EPR	CIA	[40]
	Dexamethasone	RGD-PEG liposomes	100	Endothelials	AIA	[46]
DMARDs	Methotrexate	Stealth-type polymeric nanoparticles	51–116	EPR	AIA	[41]
	Methotrexate	PEGylated liposomes	210–260	EPR	AIA	[42]
	Clodronate	Liposomes	120–160	Macrophages	AIA	[52]
Biological agents	Etanercept	TMN complex	250	EPR	CIA	[43]
	Anakinra	Folate-chitosan DNA nanoparticles	110	Macrophages	AIA	[47]
	Tocilizumab	Hyaluronate-gold nanoparticles	64	IL-6R^+^ cells	CIA	[48]
Others inhibitor	γ-secretase inhibitor	Hyaluronan nanoparticles	255	Macrophages	CIA	[50]
	Fumagillin	Perfluorocarbon nanoparticle	250	α_V_β_3_ integrin activated cells	K/BxN mouse model	[51]

AIA: adjuvant-induced arthritis; CIA: collagen-induced arthritis; EPR: enhanced permeability and retention; PEG-PAMAM: poly(ethylene glycol) conjugates of anionic dendrimer; RGD-PEG: RGD peptide-polyethylene glycol; TMN: temperature-modulated noncovalent interaction.

**Table 3:** Application of lipid-based nanoparticles for rheumatoid arthritis.

Lipid Nanocarrier	Drugs	Mean Size (nm)	Route of Administration	In Vitro/In Vivo Studies	Reference
Liposomes	Loperamide	102	Topical	AIA	[54]
	Lactoferrin	-	SC	AIA	[63]
Niosomes	Ursolic acid	665	Topical	AIA	[65]
	Luteolin	534–810	Topical	AIA	[66]
Ethosomes	Capsaicin	217–295	Topical	Rat skin	[68]
	Tetrandrine	78	Topical	Rat skin	[69]
Transfersomes	Capsaicin	94	Topical	AIA	[73]
	Celecoxib	100	Topical	Rat skin	[72]
	Piroxicam	655	Topical	Porcine skin	[74]
SLN	Piperine	128	Oral and topical	AIA	[79]
	Actarit	241	IV	Patients	[80]
	Methotrexate	250	-	THP-1 cells	[81]
	Tripterygium	116	Oral	AIA	[82]
	Curcumin	134	Oral	AIA	[83]
NLC	Methotrexate	181	Topical	AIA	[87]
	Flurbiprofen	214	Topical	Carrageenan-induced rat paw edema	[88]
lipid nanoemusion	Methotrexate	-	IV	AIA	[89]
	Low-density lipoprotein and high-density lipoprotein	148	IV	Patients	[90]

AIA: adjuvant-induced arthritis; IV: intravenous; SC: subcutaneous; SLN: solid lipid nanoparticles; NLC: nanostructured lipid carriers.

**Table 4:** Clinical application of nanotherapeutic agents in arthritic diseases.

Patent	Lipid Nanocarrier	Advantage Function
US 20150174069 A1	Dexamethasone sodium phosphate liposome	There is about a 10% reduction in one or more symptoms of arthritis
WO 2003000190 A2	Glycosaminoglycans liposome	It provides good efficacy in treatment of osteoarthritis
CN 104688721 A	Paclitaxel liposome	The gel achieves a treatment effect and pain of a patient suffering from RA
US 20090232731 A1	Cationic liposome	It provides reduction of the infiltration of mononuclear cells into the synovial tissue, pannus development and cartilage erosion
US 20160000714	Curcumin solid lipid particles	It provides suppression of cyclooxygenase 2 (COX-2) expression
WO 2017025588 A1	Cyclosporine solid lipid particles	It prevents transcription of interleukin 2, thereby decreasing activation and proliferation of T lymphocytes.
US 8715736 B2	Nanostructured Lipid Carriers	It provides efficient skin permeation at the inflammatory site in RA
CN 102225205 B	Tripterine nanostructured lipid carrier	It provides inhibition of rheumatoid arthritis inflammation
